# RNA Toxicity and Missplicing in the Common Eye Disease Fuchs Endothelial Corneal Dystrophy*[Fn FN2]

**DOI:** 10.1074/jbc.M114.621607

**Published:** 2015-01-15

**Authors:** Jintang Du, Ross A. Aleff, Elisabetta Soragni, Krishna Kalari, Jinfu Nie, Xiaojia Tang, Jaime Davila, Jean-Pierre Kocher, Sanjay V. Patel, Joel M. Gottesfeld, Keith H. Baratz, Eric D. Wieben

**Affiliations:** From the ‡ Department of Cell and Molecular Biology, The Scripps Research Institute, La Jolla, California 92037 and; the Departments of § Biochemistry and Molecular Biology,; ¶ Health Science Research, and; ‖ Ophthalmology, Mayo Clinic, Rochester, Minnesota 55905

**Keywords:** Alternative Splicing, Cornea, Eye, RNA Splicing, Trinucleotide Repeat Disease, Fuchs Corneal Dystrophy, RNA Foci, RNA Toxicity

## Abstract

Fuchs endothelial corneal dystrophy (FECD) is an inherited degenerative disease that affects the internal endothelial cell monolayer of the cornea and can result in corneal edema and vision loss in severe cases. FECD affects ∼5% of middle-aged Caucasians in the United States and accounts for >14,000 corneal transplantations annually. Among the several genes and loci associated with FECD, the strongest association is with an intronic (CTG·CAG)*_n_* trinucleotide repeat expansion in the *TCF4* gene, which is found in the majority of affected patients. Corneal endothelial cells from FECD patients harbor a poly(CUG)*_n_* RNA that can be visualized as RNA foci containing this condensed RNA and associated proteins. Similar to myotonic dystrophy type 1, the poly(CUG)*_n_* RNA co-localizes with and sequesters the mRNA-splicing factor MBNL1, leading to missplicing of essential MBNL1-regulated mRNAs. Such foci and missplicing are not observed in similar cells from FECD patients who lack the repeat expansion. RNA-Seq splicing data from the corneal endothelia of FECD patients and controls reveal hundreds of differential alternative splicing events. These include events previously characterized in the context of myotonic dystrophy type 1 and epithelial-to-mesenchymal transition, as well as splicing changes in genes related to proposed mechanisms of FECD pathogenesis. We report the first instance of RNA toxicity and missplicing in a common non-neurological/neuromuscular disease associated with a repeat expansion. The FECD patient population with this (CTG·CAG)*_n_* trinucleotide repeat expansion exceeds that of the combined number of patients in all other microsatellite expansion disorders.

## Teaser

**Background:** Expansion of intronic (CTG·CAG)*_n_* repeats in *TCF4* is found in most Fuchs endothelial corneal dystrophy (FECD) patients.

**Results:** RNA foci co-localizing with the splicing factor MBNL1 are found in FECD cells, and changes in mRNA splicing occur.

**Conclusion:** Trinucleotide repeat expansion in FECD is associated with RNA focus formation and missplicing.

**Significance:** RNA toxicity occurs in a disease affecting millions of patients.

## Introduction

The corneal endothelium is a non-regenerative cell monolayer on the internal surface of the cornea and is responsible for the maintenance of corneal clarity by continual deturgescence of the collagenous corneal stroma. Fuchs endothelial corneal dystrophy (FECD)^3^ is a common, inherited, corneal endothelial degeneration. After age 40, ∼5% of adults in the United States exhibit guttae, the clinical hallmark of the disease, which are microscopic but easily identifiable collagenous excrescences interspersed among the corneal endothelial cells. Mild disease is asymptomatic, whereas severe disease develops in a small proportion of patients with guttae. Advanced FECD, treatable only by corneal transplantation, is characterized by extensive guttae, endothelial cell loss, and vision loss due to stromal edema. Vision loss in FECD is the most frequent indication for allogeneic corneal transplantation in the United States, responsible for >14,000 grafts annually (1). FECD also poses a risk factor for irreversible corneal edema after cataract extraction (2, 3), and it may be a contraindication to refractive error-correcting procedures such as LASIK (laser-assisted *i**n* *s**itu* keratomileusis).

The influence of genetic factors on FECD is well recognized, and variants in several genes have been associated with a small proportion of FECD patients. A genome-wide association study by Baratz *et al.* (4) identified the most reproducible and robust SNP marker for FECD available at this time, the SNP rs613872, which is located in an intron of the *TCF4* (transcription factor 4) gene on chromosome 18. This association has now been replicated repeatedly, and subsequent investigation has revealed its tight linkage to a (CTG·CAG)*_n_* trinucleotide repeat (TNR) expansion in a different intron of the *TCF4* gene (5, 6) A repeat length longer than 150 nucleotides in leukocyte DNA is highly predictive of disease, so this TNR is a prime candidate for being pathogenic in this autosomal dominant, late-onset, degenerative disease (5, 7). The location of the repeat in an intron raised the possibility that RNA toxicity might play a role in the pathogenesis of this common autosomal dominant disorder, as it does in several of the relatively rare neurodegenerative and neuromuscular repeat expansion diseases, such as myotonic dystrophy types 1 and 2 (DM1 and DM2) (8), fragile X-associated tremor/ataxia syndrome (FXTAS) (9), and *C9ORF72*-associated amyotrophic lateral sclerosis and frontotemporal dementia (*C9ORF72* ALS/FTD) (10). In each of these diseases, expanded microsatellite DNA sequences are found in noncoding regions of various genes, including 5′-UTRs (FXTAS), introns (DM2 and *C9ORF72* ALS/FTD), and 3′-UTRs (DM1) (reviewed in Ref. 11), and these repetitive elements are transcribed into toxic gain-of-function RNAs. In the case of DM1, the (CTG·CAG)*_n_* repeats in the 3′-UTR of the serine/threonine protein kinase gene *DMPK* (dystrophia myotonica protein kinase) are transcribed into poly(CUG) mRNA, which disrupts normal cellular processes at the level of mRNA processing by sequestering the splicing regulator MBNL1 (muscleblind-like 1−) (8, 12). Such protein-RNA complexes can be visualized in DM1 cells as nuclear RNA foci (reviewed in Ref. 12). Because MBNL1 also binds 3′-UTRs, misregulation of alternative polyadenylation has been described in DM1 (13). RNA foci, protein sequestration and consequent pathological defects have been noted in DM2, FXTAS, and *C9ORF72* ALS/FTD (11). Recent studies also point to the possibility of translation of the repeat RNAs into toxic protein species (14). Our results demonstrate that RNA toxicity occurs in the corneal endothelia of FECD patients. We report the first instance of RNA toxicity and splicing defects in a very common disease, where the number of FECD patients alone greatly exceeds that of all patients with other microsatellite disorders worldwide. Our results also suggest potential novel therapeutic approaches to the treatment of FECD.

## EXPERIMENTAL PROCEDURES

### 

#### 

##### FECD Patients

Participants were recruited after informed consent from the cornea service of the Department of Ophthalmology at the Mayo Clinic. FECD severity was graded using a modified Krachmer scale (grade 0 (no guttae) to grade 6 (confluent guttae with corneal edema)) (15, 16). Corneal endothelial tissue was obtained by stripping an 8-mm diameter portion of the central Descemet membrane with the attached corneal endothelial cell monolayer at the time of routine corneal endothelial transplantation and immediately frozen. Skin biopsies were obtained using standard techniques.

##### Control Corneas

Normal human corneas were obtained as corneoscleral buttons from the Minnesota Lions Eye Bank or as fresh surgical enucleation specimens for pathology not involving the anterior segment of the eye. Corneal endothelial tissue was excised by bluntly stripping the central 9–10-mm diameter Descemet membrane with the attached endothelial cell monolayer and immediately frozen.

##### RNA Isolation from the Corneal Endothelium

RNA was extracted using QIAzol and QIAcube (both from Qiagen, Valencia, CA). As part of the QIAcube process, RNA was treated with DNase and then eluted in 30 μl of RNase-free 1× Tris-EDTA buffer. The integrity of the RNA was assessed by measurement of the RNA integrity number of each sample using an Agilent Bioanalyzer (17).

##### Isolation of Primary Fibroblasts and Cell Culture

Biopsies were performed at the Mayo Clinic following an approved human subject protocol. Dermal explant cultures were established from Dispase-treated skin biopsies on fibronectin underneath a glass coverslip with fibroblast medium after 5–7 days. After establishment, primary dermal fibroblasts were cultured as normal fibroblasts. Control unaffected fibroblasts (GM08333) were obtained from the Coriell Institute for Medical Research (Camden, NJ). Fibroblasts were grown at 37 °C and 5% CO_2_ with 10% FBS in minimum medium, 2 mm glutamine, 1% nonessential amino acids, 20 mm HEPES, and 1% antibiotic/antimycotic (all from Invitrogen).

##### Conventional PCR

Genomic DNA was purified by isopropyl alcohol precipitation (18). For detection of CTG·CAG TNR length in the *TCF4* gene, Platinum *Pfx* DNA polymerase (Invitrogen) was used for conventional PCR according to the manufacturer. 40 ng of genomic DNA and 0.3 μm primers 5-TCF-Fuchs 2 and 3-TCF-Fuchs 2 were used in 20-μl reactions cycled through the following conditions: 94 °C denaturation for 20 s, 60 °C annealing for 30 s, and 68 °C extension for 3 min for 40 cycles with a 5-min initial denaturation and a 10-min final extension. PCRx enhancer solution gave a final concentration of 1.0×. PCR products with CTG·CAG TNRs from the *TCF4* locus contain 238 bp of non-repeat sequences, and TNR number estimations were adjusted accordingly. 5-TCF-Fuchs 2, 5′-TGCCAGATGAGTTTGGTGTAAGATGCA-3′; and 3-TCF-Fuchs 2, 5′-CAACAAGCAGAAAGGGGGCTGCAA-3′.

##### FISH

Fibroblasts and corneal tissue on coverslips were washed with PBS once and fixed in 4% paraformaldehyde in PBS for 30 min at room temperature. After fixation, cells were washed twice with PBS and stored in 70% ethanol at 4 °C. Cells were rehydrated in 50% formamide and 2× SSC for 5 min at room temperature. The cells were then hybridized overnight at 37 °C in 100 μl of a mixture containing 10% dextran sulfate, 2 mm vanadyl-ribonucleoside complex, 0.2% BSA, 100 μg of yeast tRNA, 2× SSC, 50% formamide, and 1.2 μg of Cy3-(CAG)_10_ probe. After hybridization and washing, cells were stained with Hoechst 33342 (1:200 dilution) for 30 min at room temperature and mounted on the slide using ProLong Gold antifade reagent. The Cy3 signal was acquired at a magnification of ×63 on a Zeiss LSM 710 laser scanning confocal microscope.

##### FISH/Immunofluorescence

After hybridization with the Cy3-(CAG)_10_ probe, the corneal endothelial layer was permeabilized with fresh PBS containing 0.5% Triton X-100 for 10 min. Corneal cells were then incubated with anti-MBNL1 antibody (1:100 in PBS; sc-47740, Santa Cruz Biotechnology) for 1 h at room temperature and with a secondary antibody conjugated with Alexa Fluor 488 (1:500 in PBS; A11001, Invitrogen) at room temperature for 30 min. Following incubation, corneal endothelial cells were washed with PBS, stained with Hoechst 33342, and mounted on a microscope slide as described above.

##### RT-PCR Splicing Assays

RNA was extracted using QIAzol and QIAcube. As part of the QIAcube process, RNA was treated with DNase and then eluted in 30 μl of RNase-free 1× Tris/EDTA buffer. 2 ng of total RNA was used to prepare cDNA using an iScript cDNA synthesis kit (Bio-Rad). RT-PCR was performed using a Platinum PCR SuperMix High Fidelity kit (Invitrogen) and primer sets described previously (19, 20). Products were analyzed by electrophoresis on 2% agarose gels and stained with ethidium bromide.

##### RNA-Seq

RNA libraries were prepared using TruSeq RNA Sample Prep Kit v2 (Illumina, San Diego, CA). Briefly, poly(A) mRNA was purified from total RNA using oligo(dT) magnetic beads, fragmented at 95 °C for 8 min, eluted from the beads, and primed for first-strand cDNA synthesis using SuperScript III reverse transcriptase and random primers (Invitrogen). Second-strand cDNA synthesis was performed using DNA polymerase I and RNase H, and double-stranded cDNA was purified using a single AMPure XP bead cleanup step (Agencourt, Danvers, MA). The cDNA ends were repaired and phosphorylated using Klenow fragment, T4 polymerase, and T4 polynucleotide kinase, followed by a single AMPure XP bead cleanup step. The blunt-ended cDNAs were modified to include a single 3′-adenylate residue using Klenow exo^−^ (3′ to 5′ exo^−^), and paired-end DNA adaptors (Illumina) with a single T base overhang at the 3′-end were ligated to the A-tailed cDNA population. Unique indexes, included in the standard TruSeq kits (12-Set A and 12-Set B), were incorporated at the adaptor ligation step for multiplex sample loading on the flow cells. The resulting constructs were purified by two consecutive AMPure XP bead cleanup steps and enriched by 12 cycles of PCR using primers included in TruSeq RNA Sample Prep Kit v2. Libraries were loaded onto paired-end flow cells at concentrations of 8–10 pm to generate cluster densities of 700,000/mm^2^ using cBot and cBot Paired-end Cluster Kit v3 (Illumina) following the manufacturer's standard protocol. The flow cells were sequenced as 51 × 2 paired-end reads on an Illumina HiSeq 2000 system using TruSeq SBS Sequencing Kit v3 and SCS v1.4.8 data collection software. The base calling, base conversion, and sample de-multiplexing were preformatted with CASAVA v1.8.2 and OLB v1.9 software (Illumina). The paired-end FASTQ format sequence was then passed through MAP-RSeq (Mayo Analysis Pipeline for RNA Sequencing) (19), which uses TopHat v.2.0 (21) for alignment (against hg19), gene expression analysis, and fusion detection. Alignment files from MAP-RSeq were loaded into Bioconductor, and splice forms for the samples were quantified using CASPER v1.9.0 software (22). Maximum likelihood estimates of the relative abundances of known transcripts were obtained using the CASPER algorithm. Genes that were expressed (total gene read count across all samples of >35,000) and had more than one alternative splice isoform were used for differential isoform analysis. Group comparisons of isoforms between control and FECD patients with repeat expansion samples was performed using limma, an R package.

## RESULTS

### 

#### 

##### Transcription of the (CTG·CAG)_n_ TNR in TCF4

**FIGURE 1.**
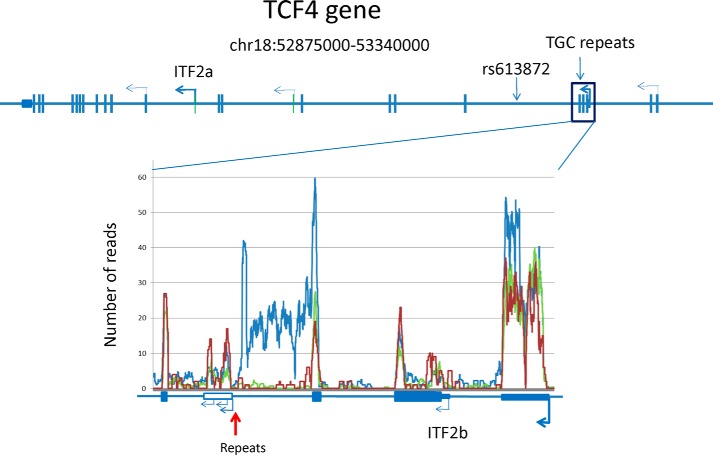
**Expression of the *TCF4* gene in the corneal endothelium.** Transcription start sites utilized in the corneal endothelium (*bent arrows*) are shown on a diagram of the intron-exon structure of the human *TCF4* gene. An expanded view of the intron-exon structure in the vicinity of the TNR (*vertical red arrow*) is shown below. A coverage plot of the average number of RNA-Seq reads from four FECD patients with a repeat expansion (*blue*), one patient without a repeat expansion (*red*), and three control samples (*green*) is aligned with the expanded gene structure diagram.

The *TCF4* gene spans 437 kb on chromosome 18 and gives rise to as many as 48 different transcripts from >20 mutually exclusive transcription start sites (23). We used RNA-Seq to determine whether stable *TCF4* intronic RNA can be detected in corneal endothelial samples. Fig. 1 provides a coverage plot of the average number of RNA-Seq reads for corneal endothelia from four FECD patients with a repeat expansion (*blue*), one patient without a repeat expansion (*red*), and three unaffected individuals (*green*). The CTG·CAG TNRs are located in an intron just downstream of a major upstream promoter used in the corneal endothelium, and the repeats are actively transcribed in both patients and controls. However, the RNA-Seq data from corneal endothelial samples also suggest that sequences from the intron containing the repeats preferentially accumulate in samples from patients with repeat expansions (compare *blue trace* with *red* and *green traces*). Clearly, transcripts upstream of the repeats predominate in the FECD samples. Note that RNA-Seq data cannot be interpreted for the repeat region, as there are numerous sites in the genome with CTG·CAG TNRs, hindering the ability to uniquely map sequences containing the repeat expansion.

##### RNA Foci in Cells from FECD Patients

**FIGURE 2.**
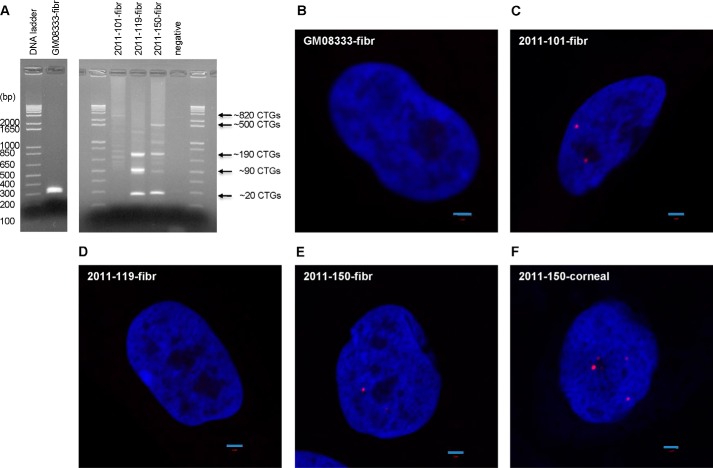
**Analysis of CTG·CAG TNR lengths and CUG RNA foci.** *A*, PCR analysis of *TCF4* CTG·CAG TNR length in the fibroblast (*fibr*) line and FECD patient fibroblasts. DNA length markers are shown in base pairs. GM08333 indicates control fibroblasts without a repeat expansion; 2011-101, 2011-119, and 2011-150 are FECD fibroblasts with repeat expansions. *negative*, no DNA control. CTG·CAG TNR length is indicated to the *right. B*, CUG RNA foci in the control fibroblast line (GM08333). *C*, CUG RNA foci in FECD patient fibroblast 2011-101. *D*, CUG RNA foci in FECD patient fibroblast 2011-119. *E*, CUG RNA foci in FECD patient fibroblast 2011-150. *F*, CUG RNA foci in FECD patient corneal tissue 2011-150. *Scale bars* = 2 μm.

To shed light on the possible mechanism by which expanded CTG·CAG TNRs might induce FECD, we derived several fibroblast cell lines from FECD patient skin biopsies. FECD fibroblasts have similar growth rates and cell morphology as normal fibroblasts. Genomic DNA extraction and PCR with primers flanking the repeats of *TCF4* revealed that the CTG·CAG TNRs are remarkably unstable in fibroblasts (Fig. 2*A*). Fibroblasts from an unaffected individual (GM08833) exhibit a single PCR product, reflecting ∼20 repeats (Fig. 2*A*, *left lanes*). FECD 2011-101 is homozygous for the expansion, whereas FECD 2011-119 and 2011-150 are heterozygous with one normal-length *TCF4* allele (∼20 repeats). In all three lines we detected multiple PCR bands, indicating that the CTG·CAG TNRs are unstable in fibroblasts and variable between patients, as shown previously for fibroblasts derived from DM1 patients (18). In contrast, the repeats are stable in leukocytes from FECD patients (data not shown, but see Wieben *et al.* (5)), similar to what is observed in leukocytes from DM1 patients (24). It is not possible at present to assess repeat instability in corneal endothelial cells, as only a single cell monolayer is obtained at the time of cornea transplantation; however, because TNR diseases are characterized by somatic instability of the repeat sequence in the affected tissues, FECD may be caused by an even larger CTG·CAG TNR expansion in the corneal endothelium. Future studies will be needed to assess this possibility.

**FIGURE 3.**
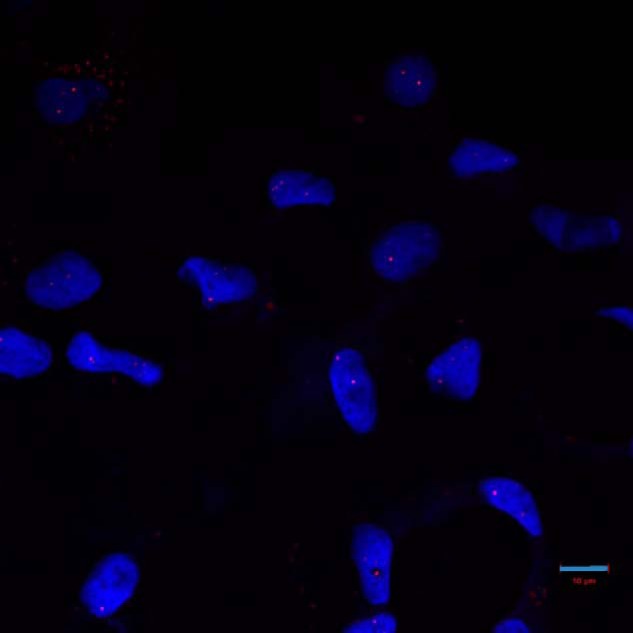
**RNA foci in FECD corneal endothelium.** CUG RNA foci, detected by FISH, are present in essentially all corneal endothelial cells from a FECD patient with expanded CTG·CAG repeats in the *TCF4* gene. *Scale bar* = 10 μm.

We examined fibroblasts and corneal endothelia from FECD patients for CUG RNA foci by FISH using a Cy3-(CAG)_10_ probe. Indeed, CUG RNA foci were detected in FECD 2011-101 and 2011-150 fibroblasts, but not in FECD 2011-119 or control fibroblasts (GM08333) (Fig. 2, *B–E*). One explanation for the absence of CUG RNA foci in FECD 2011-119 fibroblasts is that the repeat length in this line is relatively shorter than in the other two FECD fibroblast lines. In addition, we detected RNA foci in only ∼30% of the 2011-101 fibroblasts and in only ∼5% of the 2011-150 fibroblasts, in agreement with the somatic instability observed by PCR (Fig. 2*A*). These results indicate that the CTG·CAG TNR length plays an important role in CUG RNA focus formation in fibroblasts. Interestingly, we observed larger brighter CUG RNA foci in the FECD 2011-150 corneal endothelium (Fig. 2*F*), similar to what is observed in DM1 muscle (8). Moreover, almost all the cells in the FECD corneal endothelium from this patient have CUG RNA foci (Fig. 3). Quantitatively, these cells have 2.35 ± 1.14 foci per cell nucleus (from multiple images). Because we have few transplant corneas available where we also have established fibroblasts from the same patients, it is not possible at present to assess the relationship between repeat numbers and frequency of RNA foci in affected corneal endothelia. Nonetheless, these data show that the CTG·CAG TNR expansion is indeed transcribed into a stable sense-strand RNA, which causes the formation of CUG RNA foci in the affected tissue of FECD, similar to what is observed in DM1 muscle (8).

##### Co-localization of MBNL1 in RNA Foci

**FIGURE 4.**
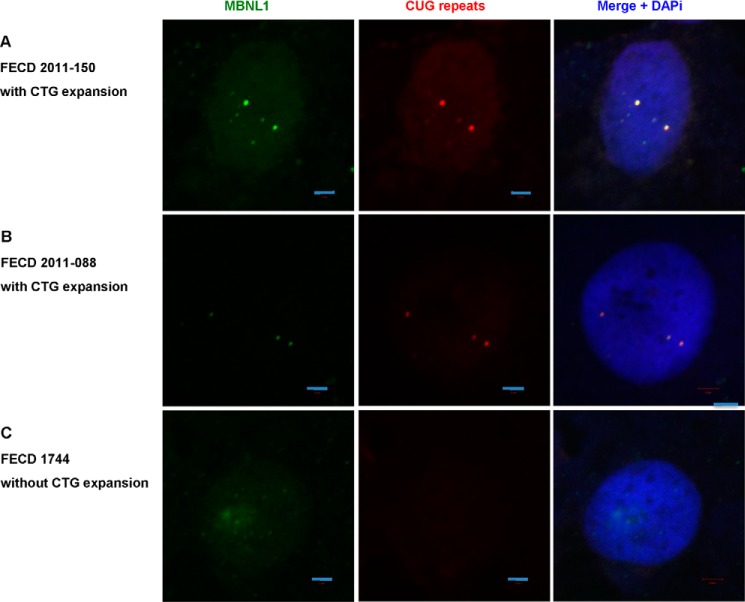
**CUG RNA foci and MBNL1 sequestration in patient corneal tissue, detected by RNA FISH and immunofluorescence staining for MBNL1.** *A*, FECD patient 2011-150 with CTG·CAG TNR expansion. *B*, FECD patient 2011-088 with CTG·CAG TNR expansion. *C*, FECD patient 1744 without CTG·CAG TNR expansion. *Scale bars* = 2 μm.

In DM1, MBNL1 sequestration by CUG RNA results in aberrant splicing of physiologically important mRNAs, such as the chloride channel ClC-1 and the insulin receptor (25, 26). To determine whether MBNL1 sequestration is also present in FECD cells, we performed FISH and MBNL1 immunofluorescence staining in FECD corneal endothelia. In patients 2011-150 and 2011-088, we clearly observed co-localization of CUG RNA foci and MBNL1 protein (Fig. 4, *A* and *B*). Both patients have a CTG·CAG TNR expansion in the *TCF4* gene. In a FECD patient with no repeat expansion (FECD 1744), we did not detect either CUG RNA foci or MBNL1 aggregation/co-localization (Fig. 4*C*).

##### TNR Expansion Alters RNA Splicing in the Corneal Endothelium

In DM1, the co-localization of poly(CUG) RNA and MBNL1 in foci leads to widespread changes in splicing patterns in affected tissues. To determine whether similar changes occur in FECD, we used CASPER analysis (22) of the corneal endothelium RNA-Seq data as an initial screen of the transcriptome-wide data. CASPER calculates the relative abundance of alternatively spliced isoforms for each gene. Using data from four corneal endothelial samples from FECD patients with repeat expansions *versus* three control samples from unaffected individuals, CASPER identified 342 genes with robust expression in the corneal endothelium that had differential expression of at least one isoform (supplemental Table 1). This analysis suggests that FECD is associated with widespread changes in splicing patterns across the genome. To confirm the CASPER results and to provide further insight into specific regulated splicing events, we also used the MISO (Mixture of Isoforms) software package (27), which provides estimates of isoform expression at the exon level. MISO quantifies the level of inclusion of a given differentially expressed exon as the “percent spliced in” (PSI or Ψ) (27). Ψ values for a given exon vary between 0 (the exon is excluded from every transcript) and 1 (the exon is included in every transcript). MISO also calculates a Bayes factor for each differential splicing event, which is a measure of the odds that there is differential inclusion of a particular exon in different samples (27).

**FIGURE 5.**
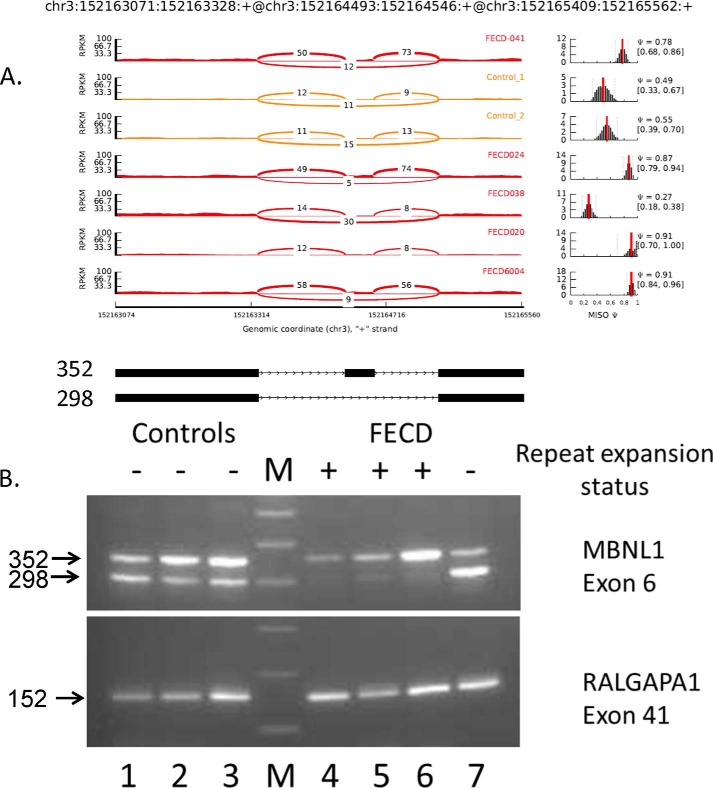
**Differential splicing of *MBNL1* in the corneal endothelium.** *A*, Sashimi plot of RNA-Seq data for *MBNL1*. The coordinates for this splicing event are shown at the *top*, and a schematic of this splicing event is shown at the *bottom*. The *main panel* shows the counts of RNA-Seq reads that span the junctions in this region of the *MBNL1* gene. The FECD samples are shown in *red*, and the control samples are shown in *yellow*. Plots of the estimated MISO Ψ values *versus* expression levels for the splicing event being examined are shown to the *right*, with the 95% confidence intervals for the Ψ estimate marked by *dashed lines. B*, RT-PCR using primers that flank selected exons was used to assess exon inclusion from *MBNL1* and *RALGAPA1* in corneal endothelial RNA samples from controls (*lanes 1–3*) and FECD patients (*lanes 4–7*). The repeat expansion status of each sample is shown (+, >50 repeats; −, <50 repeats). The size of the PCR products in base pairs is indicated to the *left. Lane M* contains size markers.

Inspection of the top differential splicing events identified by CASPER in corneal endothelial samples revealed several that have been characterized in DM1 and are known to be sensitive to MBNL1 depletion. As shown in Table 1, MBNL1-regulated events were also identified in the MISO analysis, including differential splicing of MBNL1 itself. Splicing of exon 6 of *MBNL1* (which is known to be autoregulated (28)) has a calculated average Ψ value from MISO of 0.52 in control samples. Inclusion of this exon is much more favored in FECD samples, as evidenced by the increase in the Ψ value to 0.87 (Fig. 5 and Table 1). Fig. 5*A* shows a Sashimi plot of the actual RNA-Seq reads that map to this region of the *MBNL1* gene, with the number of reads that span each part of the splice junction shown on the plots for six of the samples analyzed. The FECD samples are shown in *red*, and two of the controls are shown in *yellow*. To the right of the Sashimi plots are plots of the estimated MISO Ψ values *versus* expression levels for the splicing event being examined, with the 95% confidence intervals for the Ψ estimate marked by *dashed lines*. Interestingly, the splicing pattern for exon 6 of *MBNL1* in the corneal endothelium from an FECD patient who does not have a repeat expansion (FECD 038) resembles that of the control samples (Ψ = 0.27).

**TABLE 1:** **Selected alternative splicing events in FECD**

Gene	Chromosomal location	Function	MISO ψ				Bayes factor	Refs.
			FECD		Controls	Δψ		
			No expansion	TNR expansion				
**Genes regulated by MBNL1**								
*MBNL1*	chr3:152164493:152164546	Regulation of RNA splicing	0.27	0.87	0.52	0.35	7	19, 30, 32
*ADD3*	chr10:111892063:111892158	Cell-cell contacts	0.07	0.32	0.05	0.27	1E + 12	19, 32
*INF2*	chr14:105181621:105181677	Actin polymerization	0.73	0.24	0.85	−0.61	1E + 12	19, 32
*SORBS1*	chr10:97110966:97111133	Actin binding/focal adhesions	0.79	0.37	0.86	−0.49	1E + 12	19, 30, 47
*GNAS*	chr20:57473996:57474040	Guanine nucleotide regulatory protein	0.42	0.66	0.45	0.21	1E + 12	31
*FGFR1*	chr8:38287200:38287466	Induction of EMT	0.29	0.95	0.68	0.28	1E + 12	31
*MBNL2*	chr13:98009050:98009103	Regulation of RNA splicing	0.01	0.39	0.03	0.36	1E + 12	19, 30

**Differential splicing events also found in DM1**								
*VEGFA*	chr6:43749693:43749824	Induction of EMT	0.52	0.80	0.60	0.20	1E + 12	30
*VPS39*	chr15:42484264:42484296	Vesicle trafficking, TGF-β signaling	0.76	0.16	0.76	−0.60	41.43	30
*AKAP13*	chr15:86201768:86201821	Rho signaling	0.76	0.57	0.80	−0.22	1E + 12	29
*NFIX*	chr19:13189427:13189549	Transcription factor	0.79	0.94	0.74	0.20	1E + 12	30
*SOS1*	chr2:39216411:39216455	Receptor signaling	0.92	0.44	0.81	−0.38	1E + 12	30

**Additional differential splicing events in EMT-related genes**								
*CSNK1G3*	chr5:122941033:122941056	Wnt/β-catenin signaling	0.41	0.47	0.73	−0.26	9562.57	33
*PPFIBP1*	chr12:27829997:27830029	Focal adhesions	0.74	0.18	0.57	−0.39	1E + 12	33, 48
*STX2*	chr12:131280665–131,280,540	Epithelial cell morphogenesis	0.38	0.60	0.35	0.26	1E + 12	33
*ITGA6*	chr2:173366500:173366629	Cell-matrix adhesion	0.65	0.14	0.65	−0.51	1E + 12	34, 49

**Other notable differential splicing events**								
*ARHGEF40*	chr14:21555479:21555622	Rho signaling	0.94	0.54	0.87	−0.33	1E + 12	53
*NUMA1*	chr11:71723941:71727306	Nuclear matrix	0.79	0.24	0.76	−0.51	1E + 12	
*PPHLN1*	chr12:42778742:42778798	Barrier formation	0.84	0.56	0.88	−0.32	1E + 12	50
*USMG5*	chr10:105155503:105155789	Mitochondrial ATP synthase component	0.84	0.75	0.32	0.43	1E + 12	

Because this *MBNL1* differential splicing event was not included in the most significant differential splicing events identified by CASPER, we performed RT-PCR to confirm splicing differences between FECD samples with repeat expansions and controls. These results are shown in Fig. 5*B* and confirm the preferential inclusion of exon 6 in FECD samples from patients with TNR expansions. *Lanes 1–3* show that two strong PCR products with sizes of 352 bp and 298 bp are produced from control samples. This contrasts markedly with the pattern of RT-PCR products produced from RNA prepared from the corneal endothelia of FECD patients with TNR expansions. For these three samples (*lanes 4–6*), the 352-bp product that retains exon 6 remains strong, but very little of the 298-bp exclusion product is detected. This result confirms the conclusion from MISO that exon 6 is preferentially included in samples from patients with repeat expansions. A sample from an FECD patient who does not have a repeat expansion is show in *lane 7*. As indicated by the low Ψ value for this splicing event from MISO, the 298-bp exclusion product is much stronger in this sample, as it is in controls. This finding is consistent with the hypothesis that the increased inclusion of this exon occurs only when MBNL1 is sequestered in RNA foci created from transcribed repeat sequences.

RT-PCR results for exon 41 of *RALGAPA1* are also shown in Fig. 5*B*. This splicing event is known not to be responsive to changes in MBNL1 levels. In this case, the pattern of splicing is the same for all samples regardless of FECD status.

**FIGURE 6.**
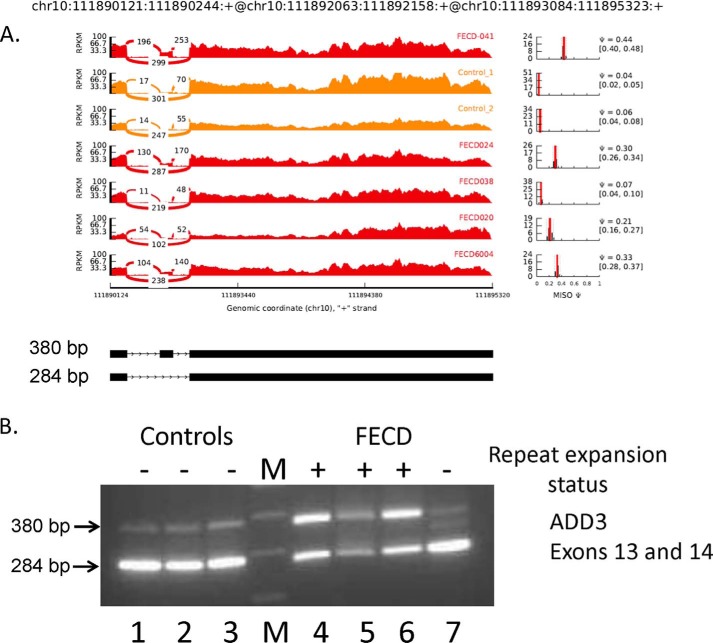
**Differential splicing of *ADD3* in the corneal endothelium.** *A*, Sashimi plot of RNA-Seq data for *ADD3*. The coordinates for this splicing event are shown at the *top*, and a schematic of this splicing event is shown at the *bottom*. The *main panel* shows the counts of RNA-Seq reads that span the junctions in this region of the *ADD3* gene. The FECD samples are shown in *red*, and the control samples are shown in *yellow*. Plots of the estimated MISO Ψ values *versus* expression levels for the splicing event being examined are shown to the *right*, with the 95% confidence intervals for the Ψ estimate marked by *dashed lines. B*, RT-PCR using primers that flank the exons shown was used to assess exon inclusion from *ADD3* in corneal endothelial RNA samples from controls (*lanes 1–3*) and FECD patients (*lanes 4–7*). The repeat expansion status of each sample is shown (+, >50 repeats; −, <50 repeats). The size of the PCR products in base pairs is indicated to the *left. Lane M* contains size markers.

**FIGURE 7.**
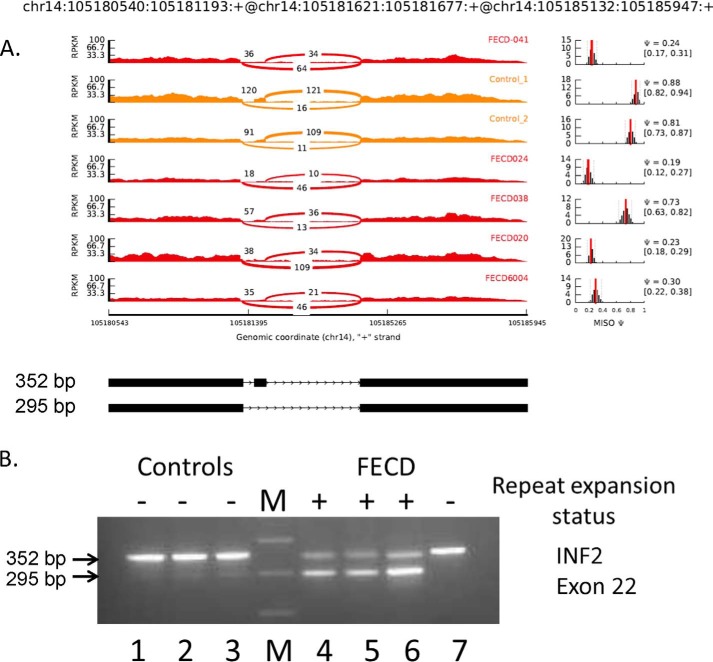
**Differential splicing of *INF2* in the corneal endothelium.** *A*, Sashimi plot of RNA-Seq data for *INF2*. The coordinates for this splicing event are shown at the *top*, and a schematic of this splicing event is shown at the *bottom*. The *main panel* shows the counts of RNA-Seq reads that span the junctions in this region of the *INF2* gene. The FECD samples are shown in *red*, and the control samples are shown in *yellow*. Plots of the estimated MISO Ψ values *versus* expression levels for the splicing event being examined are shown to the *right*, with the 95% confidence intervals for the Ψ estimate marked by *dashed lines. B*, RT-PCR using primers that flank the exons shown was used to assess exon inclusion from *INF2* in corneal endothelial RNA samples from controls (*lanes 1–3*) and FECD patients (*lanes 4–7*). The repeat expansion status of each sample is shown (+, >50 repeats; −, <50 repeats). The size of the PCR products in base pairs is indicated to the *left. Lane M* contains size markers.

Similar data for two additional splicing events known to be sensitive to MBNL1 depletion are shown in Figs. 6 and 7. Fig. 6 shows that inclusion of *ADD3* exon 14 is favored in FECD corneal endothelial samples (average Ψ = 0.32), but this exon is almost always excluded in samples from controls (average Ψ = 0.05). Once again, the splicing pattern for exon 14 of *ADD3* in the corneal endothelium from an FECD patient who does not have a repeat expansion (FECD 038) resembles that from the control samples (Ψ = 0.07). RT-PCR analysis of this splicing event (Fig. 6*B*) confirmed the increased inclusion of exon 14 (380-bp band) in FECD samples from patients who have TNR expansions. In both the control samples and the FECD sample that lacks a repeat expansion, the 284-bp band representing the PCR product that excludes exon 14 predominates.

The splicing of exon 22 of *INF2* is also known to be regulated by MBNL1 (19). For *INF2*, the FECD samples show preferential exclusion of exon 22 compared with the controls (average ΔΨ = −0.61) (Fig. 7). Once again, this RNA-Seq result was confirmed by RT-PCR. The 352-bp inclusion product predominates in controls, but the 295-bp exclusion product is favored in the FECD samples from patients without repeat expansions (Fig. 7*B*).

Both the *ADD3* (NM_016824) and *INF2* (NM_022489) transcripts were identified as being differentially spliced between the control and FECD-with-repeat-expansion groups by CASPER as well, with *p* values of 0.0001 and 0.0002, respectively (supplemental Table 1). Table 1 identifies four additional instances of differentially spliced exons known to be sensitive to MBNL1 depletion (*SORBS1*, *GNAS*, FGFR1, and *MBNL2*) that were identified by both MISO and CASPER. In each case, the Ψ values from a patient who lacked a TNR expansion were much closer to those from control samples than to those from other FECD samples.

*MBNL1*, *MBNL2*, and *SORBS1* splicing is known to be differentially regulated in DM1. Table 1 details another five splicing events found in corneal endothelial samples from patients with TNR repeat expansions that are also known to be differentially spliced in DM1 (*VEGFA*, *VPS39*, *AKAP13*, *SOS1*, and *NFIX*), emphasizing the similarity in cellular phenotype to a known TNR expansion disease (29–32).

Differential splicing of *VEGFA*, *SORBS1*, *ADD3*, *INF2*, and *FGFR1* transcripts has also been described as part of the reprogramming of gene expression that accompanies epithelial-to-mesenchymal transition (EMT) (19, 33, 34). Because EMT has been proposed to play a role in the pathogenesis of FECD (35), we surveyed the differential splicing data from FECD samples for other alternative splicing events that have been associated with EMT. Table 1 lists four additional splicing events from the RNA-Seq data in FECD that also occur during EMT. In the case of *CSNK1G3*, the Ψ value of an FECD sample from a patient without a TNR expansion (Ψ = 0.41) is more similar to the Ψ values of the other FECD samples (Ψ_avg_ = 0.47) compared with the control samples (Ψ_avg_ = 0.73). Work in other systems has shown that this splicing event is responsive to depletion of PTBP1, but is relatively insensitive to depletion of MBNL1 (19).

Additional differential splicing events potentially relevant to proposed mechanisms for FECD pathogenesis are presented in Table 1. Each of these genes is expressed at significant levels in the corneal endothelium, and splicing patterns vary significantly between FECD and control samples.

## DISCUSSION

### 

#### 

##### FECD Is a Common TNR Expansion Disease

Our results suggest that the expansion of a CTG·CAG repeat in the *TCF4* gene contributes to FECD through a mechanism that involves sequestration of MBNL1 in RNA foci. This transcribed TNR lies 43 kb upstream of the rs613872 SNP, which was significantly associated with the risk for FECD in the genome-wide association study reported by Baratz *et al.* (4). In agreement with a significant body of previous work (reviewed in Refs. 35 and 36) supporting the conclusion that the genetics of FECD involve locus heterogeneity, ∼70–80% of FECD patients in the United States have a repeat expansion in leukocyte DNA (5, 6). Previous work has shown that expansion beyond 50 repeats is associated with FECD, but it was not clear how this expansion might lead to disease (5). Both the autosomal dominant genetics and the knowledge that haploinsufficiency at the *TCF4* locus leads to a severe congenital disease (Pitt-Hopkins syndrome) argue that the pathogenesis of FECD is likely to involve gain-of-function mechanisms. The recent discovery of repeat-associated non-ATG translation in several of the RNA toxicity microsatellite disorders (14) also raises the possibility that the pathogenesis of FECD may also be due to small peptides generated from the poly(CUG)*_n_* RNA.

RNA toxicity, first characterized in the context of DM1, is now well established as an important mechanism for pathogenesis in other microsatellite diseases. In the case of myotonic dystrophy, the link between similar poly(CUG) RNA, MBNL1 sequestration, missplicing, and DM1 pathogenesis has been firmly established (8). Transgenic mouse models expressing poly(CUG) RNAs (37, 38) recapitulate skeletal muscle wasting and myotonia along with MBNL1 sequestration and missplicing of essential muscle transcripts. Similarly, knock-out of *MBNL1* is sufficient to recapitulate muscle degeneration and RNA-splicing defects, whereas overexpression of *MBNL1* prevents poly(CUG)-induced myotonia, myopathy, and splicing defects in DM1 mice (39). Finally, disruption of MBNL1-poly(CUG) RNA complexes with either morpholino-oligonucleotides or small molecules is sufficient to reverse pathological findings in DM1 mouse (25) or cell (40) models. RNA toxicity is not limited to DM1, but has also been implicated in DM2, FXTAS, and *C9ORF72* ALS/FTD (11). Interestingly, the morphology of RNA foci differs between diseases caused by different repeat sequences, but FECD and DM1 foci appear to be identical in terms of their compactness and distribution in the cell nucleus. This is likely due to the fact that both are caused by the same DNA repeat sequence.

We propose that similar mechanisms are operative in FECD and DM1. The selective abundance of poly(CUG) RNA foci in FECD corneal endothelial cells compared with a non-target cell type (fibroblasts) (Figs. 2 and 3) suggests that *TCF4* poly(CUG) transcripts predominantly accumulate in the corneal endothelium, leading to FECD pathogenesis. This observation of tissue-specific RNA accumulation may reflect the corneal endothelium's exposure to an environment prone to oxidative stress and the potential for oxidative stress to increase somatic instability and further expansion of TNRs (41). Importantly, oxidative stress has been implicated previously as a factor in the pathogenesis of FECD (42).

##### Splicing Changes Provide Insight into Possible Pathogenic Mechanisms

The widespread splicing changes identified here provide some important insights into the biology of FECD. Considering the regulatory role of *TCF4* in EMT (reviewed in Refs. 43 and 44), this pathway has also been implicated in the pathogenesis of FECD (4, 35). Missense mutations in *ZEB1*, a known inducer of EMT, have been shown to cause FECD in some patients (45). Alterations in *MBNL1* splicing have also been implicated as an important factor in EMT (19, 46). Our RNA-Seq results support a possible role for this pathway in FECD in that we have identified eight additional mRNA-splicing changes in FECD RNA samples that have previously been described in large-scale studies of splicing changes during EMT. Two of these splicing changes (in *INF2* and *ITGA6*) have ΔΨ values of >0.5, suggesting that the repeat expansion can have a switch-like effect on some splicing events that influence this process. The other changes are less dramatic but could clearly have major effects on cell function over the 5 decades it takes to develop manifestations of FECD.

The *SORBS1*, *ADD3*, and *INF2* proteins all have roles in actin dynamics within cells, and ADD3 is thought to function in cell-cell adhesion. The murine ortholog of SORBS1, called ponsin, is a focal adhesion protein localized at both cell-cell and cell-matrix adherens junctions (47). PPFIBP1 and ITGA6 also have roles in cell adhesion (48, 49), and the *PPHLN1* protein is found at cell-cell junctions and is thought to have a role in barrier formation (50), highlighting the possibility that this aspect of endothelial cell biology plays a role in the pathogenesis of FECD. Rho-associated kinase inhibitors have been suggested as possible treatments for FECD (51). It is notable that ponsin isoforms are regulated by Rho GTPases (52), whereas the *ARHGEF40* and *AKAP13* proteins also have roles in Rho signaling (53). These results suggest that further studies of RNA metabolism in FECD could provide significant insight into the disease process.

Perhaps the most significant aspect of the work presented here is that a TNR expansion appears to play a key role in the development of such a common disease. Previously characterized TNR expansion diseases are both phenotypically severe and quite rare. Our work raises the possibility that other common genetic diseases might also be due to unstable repeat expansions. Furthermore, we anticipate that future work on the pathogenesis of this TNR expansion disorder will inform studies of the other rare repeat expansion diseases. Because significant progress has been made in the development of repeat-targeting therapies for DM1 (reviewed in Refs. 25 and 40), it is likely that these approaches will also be valuable for the treatment of FECD, and therapeutic trials using simple ocular delivery methods might facilitate rapid advancement of these therapies for this and potentially other diseases.

## Footnotes

3 The abbreviations used are:

FECD: Fuchs endothelial corneal dystrophy
TNR: trinucleotide repeat
DM: myotonic dystrophy type
FXTAS: fragile X-associated tremor/ataxia syndrome
*C9ORF72* ALS/FTD: *C9ORF72*-associated amyotrophic lateral sclerosis and frontotemporal dementia
EMT: epithelial-to-mesenchymal transition.

## References

[1] Eye Bank Association of America (2014) 2013 Eye Banking Statistical Report, Washington, D.C.

[2] Doors M. Berendschot T. T. Touwslager W. Webers C. A. Nuijts R. M. (2013) Phacopower modulation and the risk for postoperative corneal decompensation: a randomized clinical trial. JAMA Ophthalmol. 131, 1443–1450 24030086 10.1001/jamaophthalmol.2013.5009

[3] Yamazoe K. Yamaguchi T. Hotta K. Satake Y. Konomi K. Den S. Shimazaki J. (2011) Outcomes of cataract surgery in eyes with a low corneal endothelial cell density. J. Cataract Refract. Surg. 37, 2130–2136 21908173 10.1016/j.jcrs.2011.05.039

[4] Baratz K. H. Tosakulwong N. Ryu E. Brown W. L. Branham K. Chen W. Tran K. D. Schmid-Kubista K. E. Heckenlively J. R. Swaroop A. Abecasis G. Bailey K. R. Edwards A. O. (2010) E2-2 protein and Fuchs's corneal dystrophy. N. Engl. J. Med. 363, 1016–1024 20825314 10.1056/NEJMoa1007064

[5] Wieben E. D. Aleff R. A. Tosakulwong N. Butz M. L. Highsmith W. E. Edwards A. O. Baratz K. H. (2012) A common trinucleotide repeat expansion within the transcription factor 4 (*TCF4*, E2-2) gene predicts Fuchs corneal dystrophy. PLoS ONE 7, e49083 23185296 10.1371/journal.pone.0049083 PMC3504061

[6] Mootha V. V. Gong X. Ku H. C. Xing C. (2014) Association and familial segregation of CTG18.1 trinucleotide repeat expansion of *TCF4* gene in Fuchs' endothelial corneal dystrophy. Invest. Ophthalmol. Vis. Sci. 55, 33–42 24255041 10.1167/iovs.13-12611 PMC3880006

[7] Zhu A. Y. Eberhart C. G. Jun A. S. (2014) Fuchs endothelial corneal dystrophy: a neurodegenerative disorder? JAMA Ophthalmol. 132, 377–378 24504267 10.1001/jamaophthalmol.2013.7993 PMC4324604

[8] Mankodi A. Urbinati C. R. Yuan Q. P. Moxley R. T. Sansone V. Krym M. Henderson D. Schalling M. Swanson M. S. Thornton C. A. (2001) Muscleblind localizes to nuclear foci of aberrant RNA in myotonic dystrophy types 1 and 2. Hum. Mol. Genet. 10, 2165–2170 11590133 10.1093/hmg/10.19.2165

[9] Hagerman R. Hagerman P. (2013) Advances in clinical and molecular understanding of the *FMR1* premutation and fragile X-associated tremor/ataxia syndrome. Lancet Neurol. 12, 786–798 23867198 10.1016/S1474-4422(13)70125-X PMC3922535

[10] DeJesus-Hernandez M. Mackenzie I. R. Boeve B. F. Boxer A. L. Baker M. Rutherford N. J. Nicholson A. M. Finch N. A. Flynn H. Adamson J. Kouri N. Wojtas A. Sengdy P. Hsiung G. Y. Karydas A. Seeley W. W. Josephs K. A. Coppola G. Geschwind D. H. Wszolek Z. K. Feldman H. Knopman D. S. Petersen R. C. Miller B. L. Dickson D. W. Boylan K. B. Graff-Radford N. R. Rademakers R. (2011) Expanded GGGGCC hexanucleotide repeat in noncoding region of *C9ORF72* causes chromosome 9p-linked FTD and ALS. Neuron 72, 245–256 21944778 10.1016/j.neuron.2011.09.011 PMC3202986

[11] Mohan A. Goodwin M. Swanson M. S. (2014) RNA-protein interactions in unstable microsatellite diseases. Brain Res. 1584, 3–14 24709120 10.1016/j.brainres.2014.03.039 PMC4174988

[12] Mankodi A. Teng-Umnuay P. Krym M. Henderson D. Swanson M. Thornton C. A. (2003) Ribonuclear inclusions in skeletal muscle in myotonic dystrophy types 1 and 2. Ann. Neurol. 54, 760–768 14681885 10.1002/ana.10763

[13] Batra R. Charizanis K. Manchanda M. Mohan A. Li M. Finn D. J. Goodwin M. Zhang C. Sobczak K. Thornton C. A. Swanson M. S. (2014) Loss of MBNL leads to disruption of developmentally regulated alternative polyadenylation in RNA-mediated disease. Mol. Cell 56, 311–322 25263597 10.1016/j.molcel.2014.08.027 PMC4224598

[14] Cleary J. D. Ranum L. P. (2014) Repeat associated non-ATG (RAN) translation: new starts in microsatellite expansion disorders. Curr. Opin. Genet. Dev. 26, 6–15 24852074 10.1016/j.gde.2014.03.002 PMC4237677

[15] Krachmer J. H. Purcell J. J. Jr. Young C. W. Bucher K. D. (1978) Corneal endothelial dystrophy. A study of 64 families. Arch. Ophthalmol. 96, 2036–2039 309758 10.1001/archopht.1978.03910060424004

[16] Repp D. J. Hodge D. O. Baratz K. H. McLaren J. W. Patel S. V. (2013) Fuchs' endothelial corneal dystrophy: subjective grading versus objective grading based on the central-to-peripheral thickness ratio. Ophthalmology 120, 687–694 23369486 10.1016/j.ophtha.2012.09.022 PMC3618600

[17] Schroeder A. Mueller O. Stocker S. Salowsky R. Leiber M. Gassmann M. Lightfoot S. Menzel W. Granzow M. Ragg T. (2006) The RIN: an RNA integrity number for assigning integrity values to RNA measurements. BMC Mol. Biol. 7, 3 16448564 10.1186/1471-2199-7-3 PMC1413964

[18] Du J. Campau E. Soragni E. Jespersen C. Gottesfeld J. M. (2013) Length-dependent CTG·CAG triplet-repeat expansion in myotonic dystrophy patient-derived induced pluripotent stem cells. Hum. Mol. Genet. 22, 5276–5287 23933738 10.1093/hmg/ddt386 PMC3842182

[19] Venables J. P. Brosseau J. P. Gadea G. Klinck R. Prinos P. Beaulieu J. F. Lapointe E. Durand M. Thibault P. Tremblay K. Rousset F. Tazi J. Abou Elela S. Chabot B. (2013) RBFOX2 is an important regulator of mesenchymal tissue-specific splicing in both normal and cancer tissues. Mol. Cell. Biol. 33, 396–405 23149937 10.1128/MCB.01174-12 PMC3554129

[20] Suenaga K. Lee K. Y. Nakamori M. Tatsumi Y. Takahashi M. P. Fujimura H. Jinnai K. Yoshikawa H. Du H. Ares M. Jr. Swanson M. S. Kimura T. (2012) Muscleblind-like 1 knockout mice reveal novel splicing defects in the myotonic dystrophy brain. PLoS ONE 7, e33218 22427994 10.1371/journal.pone.0033218 PMC3302840

[21] Kim D. Pertea G. Trapnell C. Pimentel H. Kelley R. Salzberg S. L. (2013) TopHat2: accurate alignment of transcriptomes in the presence of insertions, deletions and gene fusions. Genome Biol. 14, R36 23618408 10.1186/gb-2013-14-4-r36 PMC4053844

[22] Rossell D. Stephan-Otto Attolini C. Kroiss M. Stöcker A. (2014) Quantifying alternative splicing from paired-end RNA-sequencing data. Ann. Appl. Stat. 8, 309–330 24795787 10.1214/13-aoas687 PMC4005600

[23] Sepp M. Kannike K. Eesmaa A. Urb M. Timmusk T. (2011) Functional diversity of human basic helix-loop-helix transcription factor TCF4 isoforms generated by alternative 5′ exon usage and splicing. PLoS ONE 6, e22138 21789225 10.1371/journal.pone.0022138 PMC3137626

[24] Ashizawa T. Dubel J. R. Harati Y. (1993) Somatic instability of CTG repeat in myotonic dystrophy. Neurology 43, 2674–2678 8255475 10.1212/wnl.43.12.2674

[25] Wheeler T. M. Lueck J. D. Swanson M. S. Dirksen R. T. Thornton C. A. (2007) Correction of ClC-1 splicing eliminates chloride channelopathy and myotonia in mouse models of myotonic dystrophy. J. Clin. Invest. 117, 3952–3957 18008009 10.1172/JCI33355 PMC2075481

[26] Savkur R. S. Philips A. V. Cooper T. A. (2001) Aberrant regulation of insulin receptor alternative splicing is associated with insulin resistance in myotonic dystrophy. Nat. Genet. 29, 40–47 11528389 10.1038/ng704

[27] Katz Y. Wang E. T. Airoldi E. M. Burge C. B. (2010) Analysis and design of RNA sequencing experiments for identifying isoform regulation. Nat. Methods 7, 1009–1015 21057496 10.1038/nmeth.1528 PMC3037023

[28] Gates D. P. Coonrod L. A. Berglund J. A. (2011) Autoregulated splicing of *muscleblind-like 1* (*MBNL1*) pre-mRNA. J. Biol. Chem. 286, 34224–34233 21832083 10.1074/jbc.M111.236547 PMC3190765

[29] Yamashita Y. Matsuura T. Shinmi J. Amakusa Y. Masuda A. Ito M. Kinoshita M. Furuya H. Abe K. Ibi T. Sahashi K. Ohno K. (2012) Four parameters increase the sensitivity and specificity of the exon array analysis and disclose 25 novel aberrantly spliced exons in myotonic dystrophy. J. Hum. Genet. 57, 368–374 22513715 10.1038/jhg.2012.37

[30] Nakamori M. Sobczak K. Puwanant A. Welle S. Eichinger K. Pandya S. Dekdebrun J. Heatwole C. R. McDermott M. P. Chen T. Cline M. Tawil R. Osborne R. J. Wheeler T. M. Swanson M. S. Moxley R. T. 3rd Thornton C. A. (2013) Splicing biomarkers of disease severity in myotonic dystrophy. Ann. Neurol. 74, 862–872 23929620 10.1002/ana.23992 PMC4099006

[31] Klinck R. Fourrier A. Thibault P. Toutant J. Durand M. Lapointe E. Caillet-Boudin M. L. Sergeant N. Gourdon G. Meola G. Furling D. Puymirat J. Chabot B. (2014) RBFOX1 cooperates with MBNL1 to control splicing in muscle, including events altered in myotonic dystrophy type 1. PLoS ONE 9, e107324 25211016 10.1371/journal.pone.0107324 PMC4161394

[32] Wang E. T. Cody N. A. Jog S. Biancolella M. Wang T. T. Treacy D. J. Luo S. Schroth G. P. Housman D. E. Reddy S. Lécuyer E. Burge C. B. (2012) Transcriptome-wide regulation of pre-mRNA splicing and mRNA localization by muscleblind proteins. Cell 150, 710–724 22901804 10.1016/j.cell.2012.06.041 PMC3428802

[33] Shapiro I. M. Cheng A. W. Flytzanis N. C. Balsamo M. Condeelis J. S. Oktay M. H. Burge C. B. Gertler F. B. (2011) An EMT-driven alternative splicing program occurs in human breast cancer and modulates cellular phenotype. PLoS Genet. 7, e1002218 21876675 10.1371/journal.pgen.1002218 PMC3158048

[34] Warzecha C. C. Carstens R. P. (2012) Complex changes in alternative pre-mRNA splicing play a central role in the epithelial-to-mesenchymal transition (EMT). Semin. Cancer Biol. 22, 417–427 22548723 10.1016/j.semcancer.2012.04.003 PMC3413750

[35] Iliff B. W. Riazuddin S. A. Gottsch J. D. (2012) The genetics of Fuchs' corneal dystrophy. Expert Rev. Ophthalmol. 7, 363–375 23585771 10.1586/eop.12.39 PMC3622272

[36] Aldave A. J. Han J. Frausto R. F. (2013) Genetics of the corneal endothelial dystrophies: an evidence-based review. Clin. Genet. 84, 109–119 23662738 10.1111/cge.12191 PMC3885339

[37] Orengo J. P. Chambon P. Metzger D. Mosier D. R. Snipes G. J. Cooper T. A. (2008) Expanded CTG repeats within the DMPK 3′ UTR cause severe skeletal muscle wasting in an inducible mouse model for myotonic dystrophy. Proc. Natl. Acad. Sci. U.S.A. 105, 2646–2651 18272483 10.1073/pnas.0708519105 PMC2268190

[38] Kanadia R. N. Johnstone K. A. Mankodi A. Lungu C. Thornton C. A. Esson D. Timmers A. M. Hauswirth W. W. Swanson M. S. (2003) A muscleblind knockout model for myotonic dystrophy. Science 302, 1978–1980 14671308 10.1126/science.1088583

[39] Chamberlain C. M. Ranum L. P. (2012) Mouse model of muscleblind-like 1 overexpression: skeletal muscle effects and therapeutic promise. Hum. Mol. Genet. 21, 4645–4654 22846424 10.1093/hmg/dds306 PMC3471398

[40] Childs-Disney J. L. Parkesh R. Nakamori M. Thornton C. A. Disney M. D. (2012) Rational design of bioactive, modularly assembled aminoglycosides targeting the RNA that causes myotonic dystrophy type 1. ACS Chem. Biol. 7, 1984–1993 23130637 10.1021/cb3001606 PMC3528830

[41] Jonson I. Ougland R. Klungland A. Larsen E. (2013) Oxidative stress causes DNA triplet expansion in Huntington's disease mouse embryonic stem cells. Stem Cell Res. 11, 1264–1271 24041806 10.1016/j.scr.2013.08.010

[42] Jurkunas U. V. Bitar M. S. Funaki T. Azizi B. (2010) Evidence of oxidative stress in the pathogenesis of Fuchs endothelial corneal dystrophy. Am. J. Pathol. 177, 2278–2289 20847286 10.2353/ajpath.2010.100279 PMC2966787

[43] Peinado H. Olmeda D. Cano A. (2007) Snail, Zeb and bHLH factors in tumour progression: an alliance against the epithelial phenotype? Nat. Rev. Cancer 7, 415–428 17508028 10.1038/nrc2131

[44] Sobrado V. R. Moreno-Bueno G. Cubillo E. Holt L. J. Nieto M. A. Portillo F. Cano A. (2009) The class I bHLH factors E2-2A and E2-2B regulate EMT. J. Cell Sci. 122, 1014–1024 19295128 10.1242/jcs.028241

[45] Riazuddin S. A. Zaghloul N. A. Al-Saif A. Davey L. Diplas B. H. Meadows D. N. Eghrari A. O. Minear M. A. Li Y. J. Klintworth G. K. Afshari N. Gregory S. G. Gottsch J. D. Katsanis N. (2010) Missense mutations in *TCF8* cause late-onset Fuchs corneal dystrophy and interact with *FCD4* on chromosome 9p. Am. J. Hum. Genet. 86, 45–53 20036349 10.1016/j.ajhg.2009.12.001 PMC2801746

[46] LeMasters K. E. Blech-Hermoni Y. Stillwagon S. J. Vajda N. A. Ladd A. N. (2012) Loss of muscleblind-like 1 promotes invasive mesenchyme formation in endocardial cushions by stimulating autocrine TGFβ3. BMC Dev. Biol. 12, 22 22866814 10.1186/1471-213X-12-22 PMC3484067

[47] Mandai K. Nakanishi H. Satoh A. Takahashi K. Satoh K. Nishioka H. Mizoguchi A. Takai Y. (1999) Ponsin/SH3P12: an l-afadin- and vinculin-binding protein localized at cell-cell and cell-matrix adherens junctions. J. Cell Biol. 144, 1001–1017 10085297 10.1083/jcb.144.5.1001 PMC2148189

[48] Norrmén C. Vandevelde W. Ny A. Saharinen P. Gentile M. Haraldsen G. Puolakkainen P. Lukanidin E. Dewerchin M. Alitalo K. Petrova T. V. (2010) Liprin β1 is highly expressed in lymphatic vasculature and is important for lymphatic vessel integrity. Blood 115, 906–909 19965622 10.1182/blood-2009-03-212274 PMC2815506

[49] Samarelli A. V. Riccitelli E. Bizzozero L. Silveira T. N. Seano G. Pergolizzi M. Vitagliano G. Cascone I. Carpentier G. Bottos A. Primo L. Bussolino F. Arese M. (2014) Neuroligin 1 induces blood vessel maturation by cooperating with the α6 integrin. J. Biol. Chem. 289, 19466–19476 24860089 10.1074/jbc.M113.530972 PMC4094057

[50] Kazerounian S. Aho S. (2003) Characterization of periphilin, a widespread, highly insoluble nuclear protein and potential constituent of the keratinocyte cornified envelope. J. Biol. Chem. 278, 36707–36717 12853457 10.1074/jbc.M303896200

[51] Koizumi N. Okumura N. Ueno M. Nakagawa H. Hamuro J. Kinoshita S. (2013) Rho-associated kinase inhibitor eye drop treatment as a possible medical treatment for Fuchs corneal dystrophy. Cornea 32, 1167–1170 23715376 10.1097/ICO.0b013e318285475d

[52] Rao P. V. Maddala R. (2009) Abundant expression of ponsin, a focal adhesion protein, in lens and downregulation of its expression by impaired cytoskeletal signaling. Invest. Ophthalmol. Vis. Sci. 50, 1769–1777 19029030 10.1167/iovs.08-2909 PMC2716002

[53] Tse S. W. Broderick J. A. Wei M. L. Luo M. H. Smith D. McCaffery P. Stamm S. Andreadis A. (2005) Identification, expression analysis, genomic organization and cellular location of a novel protein with a RhoGEF domain. Gene 359, 63–72 16143467 10.1016/j.gene.2005.06.025

